# Dose-Volume Predictors of Radiation Pneumonitis After Lung Stereotactic Body Radiation Therapy (SBRT): Implications for Practice and Trial Design

**DOI:** 10.7759/cureus.10808

**Published:** 2020-10-05

**Authors:** Vitali Moiseenko, Jimm Grimm, Ellen Yorke, Andrew Jackson, Anthony Yip, Minh-Phuong Huynh-Le, Anand Mahadevan, Kenneth Forster, Michael T Milano, Jona A Hattangadi-Gluth

**Affiliations:** 1 Radiation Medicine and Applied Sciences, University of California San Diego Moores Cancer Center, La Jolla, USA; 2 Radiation Oncology, Geisinger Health System, Danville, USA; 3 Medical Physics, Memorial Sloan Kettering Cancer Center, New York, USA; 4 Radiation Oncology, Geisinger Cancer Institute, Danville, USA; 5 Radiology Oncology, Wilmot Cancer Institute, University of Rochester, Rochester, USA

**Keywords:** stereotactic body radiation therapy, radiation pneumonitis, dose-volume-response

## Abstract

Background and purpose

Recently published HyTEC report summarized lung toxicity data and proposed guidelines of mean lung dose (MLD) <8 Gy and normal lung receiving at least 20 Gy, V_20Gy_<10-15% to avoid lung toxicity. Support for preferred use of a particular dosimetric parameter has been limited. We performed a detailed dose-volume analysis of data on radiation pneumonitis (RP) following lung stereotactic body radiation therapy (SBRT) to search for parameters showing the strongest correlation with RP.

Materials and methods

Two patient cohorts (primary and metastatic lung tumor patients) from previously reported studies were analyzed. Total number of patients was 96, and incidence of grade ≥2 RP was 13.5% (13/96). Fitting to the logistic function was performed to investigate correlation between incidence of RP and reported dosimetric and volumetric parameters. Another independent cohort was used to explore correlation between dosimetric parameters.

Results

Among normal lung parameters (MLD and reported V_x_), only MLD consistently showed significant correlation with incidence of RP. Gross tumor volume (GTV), internal target volume, planning target volume (PTV), and minimum dose covering 95% of GTV or PTV did not show statistical significance. A significant correlation between reported V_x_ and MLD was observed in all cohorts.

Conclusions

In considering tumor- and target-specific (e.g., GTV, PTV) and normal lung-specific (e.g., MLD, V_x_) metrics, MLD was the only parameter that consistently correlated with incidence of RP across both cohorts. Because SBRT planning constraints allow small normal lung volumes to receive high doses, utility of MLD is not obvious. The parallel structure of lung is one possible explanation, but correlation between dosimetric parameters obscures elucidation of the preferred or mechanistically based parameter to guide radiotherapy planning.

## Introduction

It has been 10 years since the QUANTEC (Quantitative Analyses of Normal Tissue Effects in the Clinic) reports have been published [1]. This effort has been followed by forming two more “TEC” working groups, HyTEC (Hypofractionated/High Dose per fraction), which is near completion with a number of papers published [2-4], and PENTEC (Pediatric Normal Tissue Effects in the Clinic), which is currently in progress [5]. Each working group has been assigned a similar set of tasks: to summarize available literature, to model normal tissue complication probability (NTCP) based upon pooled published data, to provide dosimetric recommendations, and to inform future studies. One of the main challenges has been to find and justify preferred metrics associated with outcomes. This task is particularly challenging not only because the published literature is inconsistent with respect to the scales used to grade toxicities as well as dosimetric parameters analyzed, but also because patient-specific data, which would allow for pooling more granular data, are rarely provided [2]. Whenever possible, hypothesis-generating studies to search for preferred metrics are needed to justify radiotherapy planning guidelines.

Stereotactic body radiation therapy (SBRT) with ablative doses has become the standard of care for patients with medically inoperable early-stage non-small cell lung cancer (NSCLC) [6]. Furthermore, the indications for SBRT have been expanded as an option for patients with early-stage, operable NSCLC [7, 8] and lung metastases [9]. Despite advances in SBRT planning and delivery, achieving the desired prescription dose must be balanced against the risk of complications, with lung toxicity being the major limiting factor [2]. Radiation pneumonitis (RP) has been reported in numerous lung SBRT studies [10], with incidence of grade 2 or higher toxicity varying from approximately 10% [11, 12] to as high as 30% [13, 14].

Robust dose-volume predictors of RP are critical in guiding clinical practice and informing lung SBRT clinical trials. Various dosimetric parameters have been tested for correlation with RP, in particular mean lung dose (MLD) [15], or dose-volume cut-offs, Vx (volume of normal lung receiving at least dose ‘x’ Gy) [16]. Commonly, statistical analyses presented in the literature only test if the values of the parameter of choice were different among patients with RP vs without RP, or if a dichotomy can be achieved based on a cut-off value of the parameter of choice. While these observations provide useful data to help guide treatment planning, they do not describe the actual normal tissue dose-volume response. To date, only a few publications have reported correlation, or lack of, between the incidence of pulmonary toxicity and lung SBRT dose-volume parameters, e.g., MLD and V20 [10, 17, 18]. Numerous studies with a goal to explore incidence of RP as a function of various dose metrics have been published for conventional fractionation [19].

Use of Vx cut-offs is appealing because this metric can be readily taken from the dose-volume histogram (DVH). However, as was pointed out in the QUANTEC report on lung toxicity, Vx cut-offs may not be universal and the most predictive values of x may depend on the treatment technique [19]. Also, because of inter-correlation among Vx parameters [20], statistics do not allow to determine the preferred single predictor which connects with the etiology of toxicity in a meaningful manner. Parameters strongly correlated with such a predictor also become predictors. Hypothesis-generating studies are a pre-requisite to select the preferred metric to guide radiotherapy planning. In this paper, based on the authors’ experience with the HyTEC effort, we present detailed dose-volume analyses of data on RP following lung SBRT from two discrete patient cohorts. We explored the incidence of RP as a function of various parameters, specifically normal lung dose-volume metrics and tumor-related metrics. In addition, we tested for correlation between such predictors in the two cohorts and an independently obtained dataset.

## Materials and methods

Patients

Two patient cohorts from previously reported studies were used, referenced below as cohorts A [21] and B [22]. These studies were selected because they provided detailed patient-specific data in a tabulated form. In brief, cohort A consisted of 66 (44 male, 22 female) patients with 71 primary (51) or metastatic (20) lung tumors, with planning target volume (PTV) ranging from 9.0 to 100.8 cc, who were treated with SBRT with prescribed doses of 40-60 Gy in 5-10 fractions over 5-12 days. Median age was 80 years (58-88 years). Prescription policies were based on the proportion of the prescribed dose covering 80% of the PTV; the median prescribed dose was 50 Gy in five fractions. Dose calculation was performed using pencil beam convolution with the Batho method used to account for tissue inhomogeneities. The authors analyzed clinical factors, specifically pulmonary emphysema, tumor location and subclinical interstitial lung disease (ILD) for association with RP (see discussed below). Cohort B consisted of 25 patients (21 male, 4 female) with primary (16), recurrent (2) or metastatic (7) lung tumors, PTV ranging from 7.5 to 239.4 cm^3^, who were treated with SBRT using a prescribed dose of 48 Gy in 4-8 fractions in 6-7 fields over 5-8 days. Median age was 77 years (73.8 ± years). Four patients presented with emphysema, three with ILD and one with chronic obstructive pulmonary disease (COPD). Planning goal was that 95% of PTV receives at least 90% of the prescribed dose. Collapsed cone convolution method was used for dose calculation. Detailed patient data have been extracted for further analysis. The normal lung parameters tabulated for cohort A were the % volumes receiving at least 5, 10, 20 and 30 Gy (V_5_,V_10_, V_20_, V_30_) and the MLD and for cohort B, the % volumes receiving at least 20, 40 and 45 Gy (V_20_, V_40_, V_45_), absolute volume in cc receiving at least 24 and 48 Gy (V_cc__24_ and V_cc__48_), and MLD. Whenever possible, subsets of patients were selected to test for significance of a parameter.

To examine potential correlations between dose-volume parameters in an independent data set, 33 consecutive patients treated with lung SBRT at the University of California San Diego (UCSD) in 2017-2018 were selected. Planning data were extracted from the Eclipse treatment planning system (Varian, Palo Alto, CA). PTV prescription dose was 40-56 Gy in 3-5 fractions. Volumetric-modulated arc therapy (VMAT) with 2-3 coplanar arcs was used, with a planning goal that at least 95% of the planning target volume (PTV) receives 100% of the prescription dose. Analytical anisotropic algorithm, accounting for tissue inhomogeneities, was used to calculate the dose. Gated treatment was used for 12 patients. In planning 4DCT data were sorted into 10 phases, 0-90 with an increment of 10, where 0 corresponds to inhale, and 50 to exhale. Phases 30-70 or 40-60 centered around exhalation were used to minimize effect of tumor motion. The remaining 21 patients received Gate 100, which means all phases from inhale to exhale are used in planning and dose delivery, and patient is monitored during treatment for breathing irregularities, for example, deep inhale or cough. Normal lung volume was defined as bilateral lung minus internal target volume (ITV). MLD, V_5_, V_13_ and V_20_ - parameters commonly used in toxicity studies following SBRT [2] - were extracted. Institutional ethics board approval was obtained to extract, analyze and present the data.

Dose-volume response analysis

Reported dose-volume parameters for normal lung and tumor-related parameters were analyzed for correlation with incidence of RP grade 2 or higher (grade 2+) and grade 3 or higher (grade 3+) using the Common Terminology Criteria for Adverse Events v5.0 (CTCAE). Specifically, we analyzed MLD and reported V_x_ for normal lung dose-volume parameters as well as gross tumor volume (GTV), ITV, and PTV for tumor-related parameters. For each parameter, fitting to the logistic function was performed; for example, if V_10_ was explored, the function was:

 $P=1/[1+exp(-4\gamma _{50}(V_{10}/V_{10,50}-1))]$

where γ_50_ is the normalized slope and V_10,50_ is the value of V10 at which 50% of patients develop RP. Maximum likelihood method was used to obtain model parameter values, γ_50_ and V_10,50_. Likelihood-profile method was used to calculate 95% confidence intervals (CI) [23]. A likelihood ratio test was used to establish if the observed dependence significantly (p < 0.05) improves on the null hypothesis assuming no dependence. To calculate likelihood value for the null hypothesis incidence was set to the value averaged over the cohort. Spearman test was used to check for correlation between dose-volume parameters. All tests were two-tailed and p < 0.05 was considered statistically significant.

## Results

**Figure 1:**
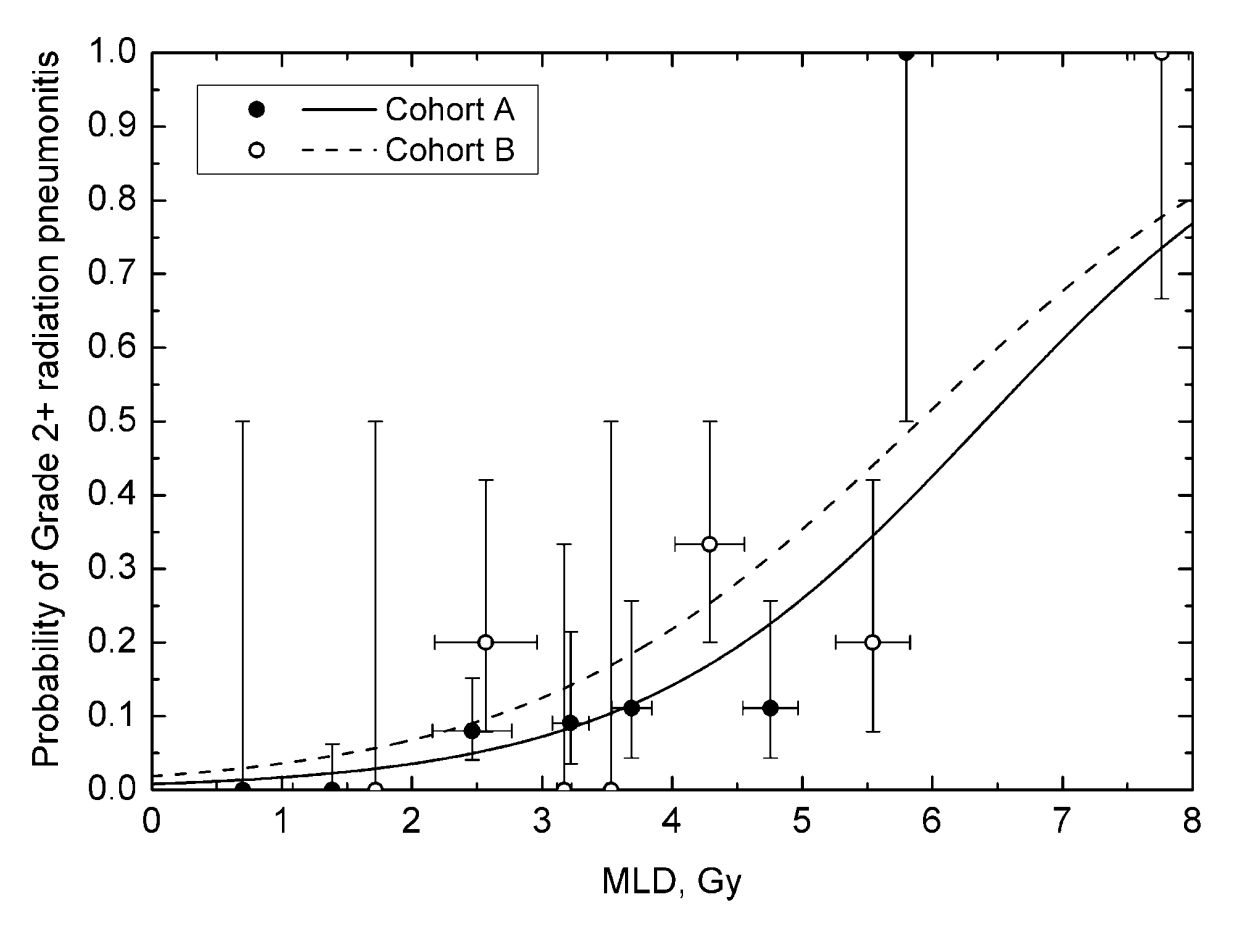
Probability of grade 2 or higher radiation pneumonitis as a function of mean lung dose (MLD) for the studied patient cohorts A and B. Vertical error bars are binomial 68% confidence intervals, horizontal error bars are standard deviations. Bin size was set to 1 Gy, except between 3 and 4 Gy where a 0.5 Gy size was used.

Total number of patients in the study was 96, 13 of whom developed RP (13.5%). This sample size and toxicity rate was similar to those published in the studies that were summarized in the HyTEC report (see supplemental table in the HyTEC report [2]). Wilcoxon rank sum test showed that MLD was significantly different, p = 0.002, in patients with vs without grade 2+ toxicity, thereby justify further analysis for dose-response. Table 1 shows the results of fitting the dose-volume data to the logistic function for both grade 2+ and 3+ toxicity in cohorts A and B. None of the tumor or target volume characteristics (GTV, ITV, PTV) showed statistical significance. Among normal lung parameters (MLD and reported V_x_), only MLD consistently showed significant correlation with incidence of RP for both cohorts (Figure 1). Specifically, MLD was statistically significant for both grade 2+ and grade 3+ in cohort A, as well as grade 2+ in cohort B. However, this significance was only marginal for grade 3+ in cohort B, p = 0.058.

**Table 1:** Grade 2+ and 3+ lung toxicity, results of fitting to the logistic model. If the likelihood-profile was flat and did not fall below the cut-off value, maximum log-likelihood minus 1.92, at either lower or upper end, confidence intervals are marked as “at limit”.

Parameter	Grade 2+			Grade 3+		
	D_50_, V_50_ or V_x,50_ (95% CI)	γ_50_ (95% CI)	p, likelihood ratio	D_50_, V_50_ or V_x,50_ (95% CI)	γ_50_ (95% CI)	p, likelihood ratio
	Cohort A [21]					
GTV, cc	78.2 (at limit)	0.68 (0.41, 1.02)	0.390	53.6 (30.5, ∞)	1.01 (0.63, 1.57)	0.098
PTV, cc	162.1 (72.3, ∞)	0.74 (0.46, 1.19)	0.431	106.8 (68.9, ∞)	1.18 (0.66, 1.95)	0.097
GTV D_95_, Gy	80.8 (at limit)	1.58 (-.25, 3.87)	0.310	91.8 (at limit)	1.73 (-0.71, 5.07)	0.479
MLD, Gy	6.4 (4.8, 88.1)	1.20 (0.58, 2.05)	0.042	6.3 (5.0, 51.8)	1.74 (0.78, 3.31)	0.037
V_5_,%	33.3 (25.2, ∞)	1.22 (0.59, 2.08)	0.038	33.8 (26.4, ∞)	1.69 (0.75, 3.22)	0.045
V_10_, %	20.4 (15.7, 69.1)	1.23 (0.65, 2.03)	0.018	20.7 (16.4, 60.3)	1.76 (0.87, 3.27)	0.016
V_20_, %	13.4 (at limit)	0.83 (0.37, 1.41)	0.294	11.1 (at limit)	1.24 (0.57, 2.29)	0.159
V_30_, %	7.8 (at limit)	0.84 (0.40, 1.42)	0.251	6.7 (at limit)	1.24 (0.58, 2.28)	0.153
	Cohort B [22]					
ITV, cc	1217.0 (at limit)	0.24 (-0.04, 0.54)	0.975	at limit	-	-
PTV, cc	368.9 (at limit)	0.28 (-0.06, 0.64)	0.752	681.7 (at limit)	0.38 (-0.01, 0.79)	0.841
PTV D_95_, Gy	49.0 (at limit)	3.38 (-3.07, 10.24)	0.332	48.6 (at limit)	5.92 (-1.15, 14.00)	0.121
MLD, Gy	5.9 (4.5, 30.5)	0.99 (0.24, 2.01)	0.037	6.8 (5.16, ∞)	1.09 (0.28, 2.22)	0.058
V_20_, %	12.1 (at limit)	0.56 (-0.01, 1.29)	0.244	13.8 (at limit)	0.72 (0.08, 1.60)	0.237
V_40_, %	5.6 (at limit)	0.39 (0.02, 0.84)	0.332	6.1 (at limit)	0.57 (0.14, 1.11)	0.240
V_45_, %	3.8 (at limit)	0.36 (0.05, 0.72)	0.303	4.7 (at limit)	0.49 (0.15, 0.91)	0.277
V_cc24_, cc	258.6 (172.3, ∞)	0.67 (0.15, 1.38)	0.076	301.1 (207.1, ∞)	0.82 (0.23, 1.71)	0.097
V_cc48_, cc	112.8 (at limit)	0.28 (0.02, 0.58)	0.597	984.7 (at limit)	0.35 (0.07, 0.68)	0.939
	Combined data					
MLD, Gy	6.1 (5.0-9.0)	1.18 (0.73-1.77)	<0.001	6.6 (5.5-10.2)	1.47 (0.90-2.27)	<0.001

**Figure 2:**
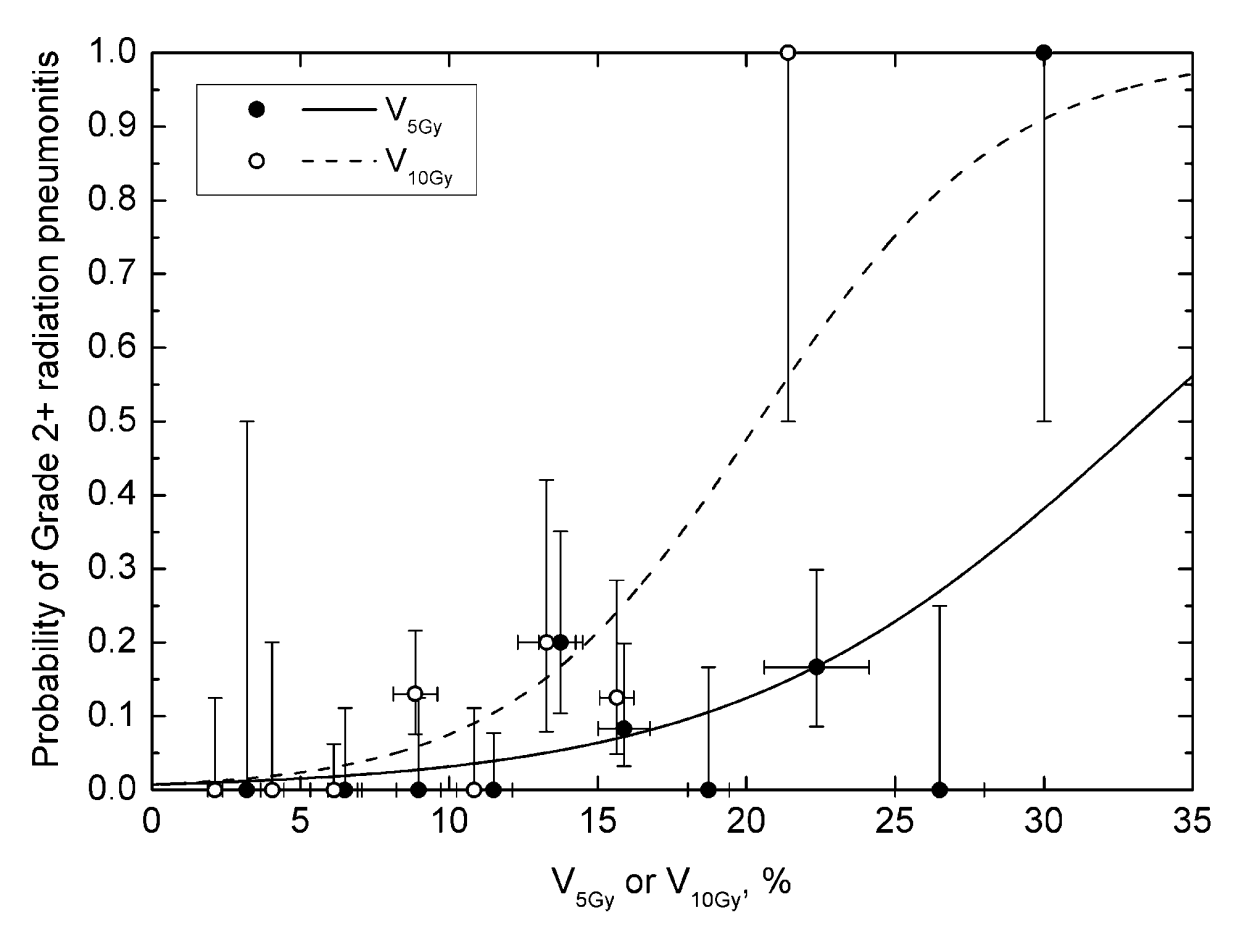
Probability of grade 2 or higher radiation pneumonitis as a function of V5 and V10, cohort A. Vertical error bars are binomial 68% confidence intervals, horizontal error bars are standard deviations. Bin size was set to 2.5%.

Based on the available dose-volume data, values of V_x_ were tested for correlation with incidence of toxicity. Only V_5_ and V_10_ in cohort A resulted in a statistically significant V_x_-response dependence (Table 1). This held for both grade 2+ and grade 3+. Values of γ_50_ and V_x,50_ for V_5_ and V_10_, cohort A, were very similar for grade 2+ and grade 3+ (Table 1). Specifically, V_5,50_ was 33.3% for grade 2+ and 33.8% for grade 3+. V_10,50_ values were 20.4% for grade 2+ and 20.7% for grade 3+. Figure 2 shows incidence of RP as a function of V_5_ and V_10_ in cohort A. No logistic fit for V_x_ was significant in cohort B.

**Figure 3:**
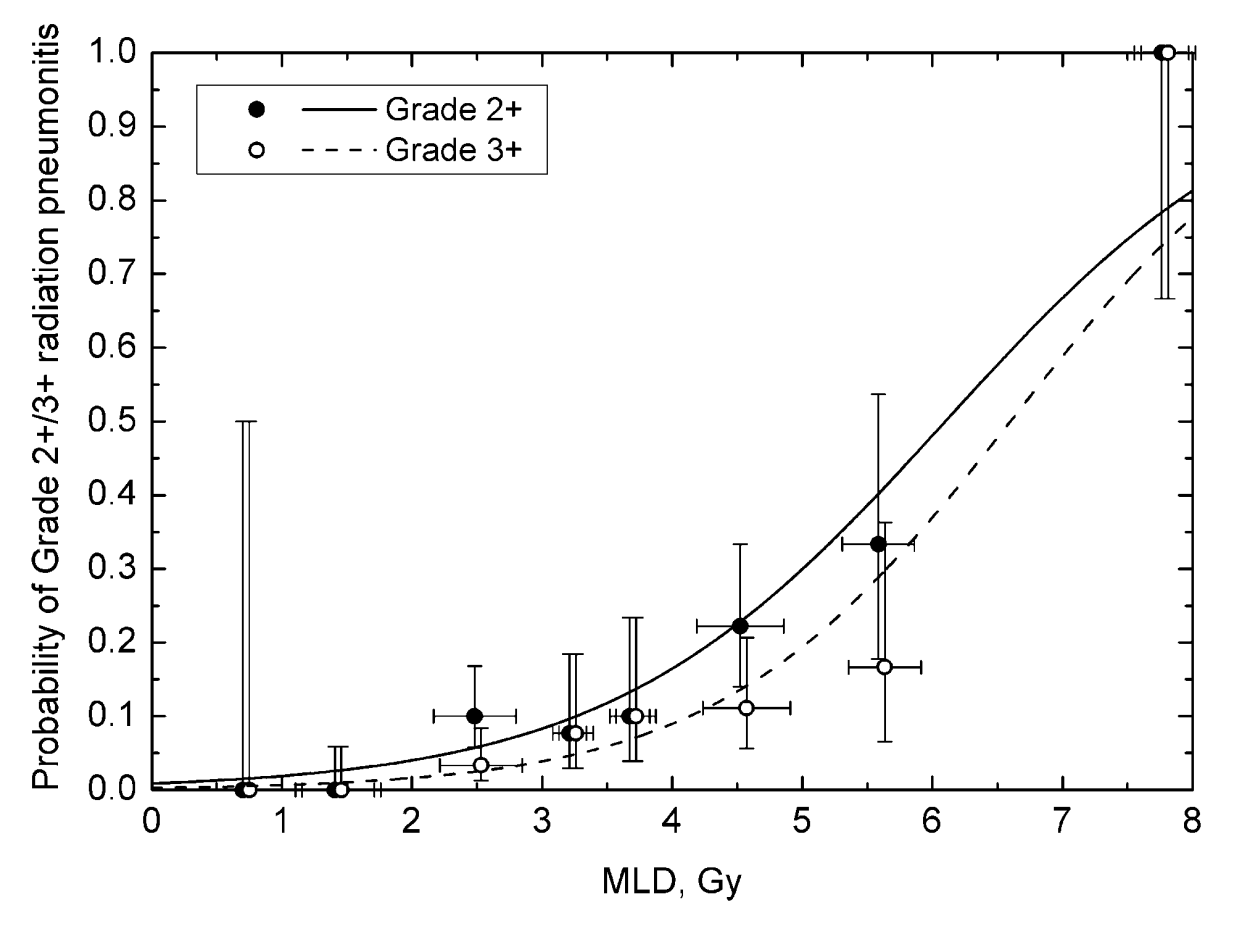
Probability of grade 2 or higher, and grade 3 or higher as a function of mean lung dose (MLD) for the combined data. Vertical error bars are binomial 68% confidence intervals, horizontal error bars are standard deviations. Bin size was set to 1 Gy, except between 3 and 4 Gy where a 0.5 Gy size was used.

MLD data from cohorts A and B were combined and statistically significant dose-response was obtained for both grade 2+ and grade 3+ toxicity. Notably, when fitting was performed separately for cohorts A and B, the values of D_50_ were very close, albeit with broad CI (Table 1). Figure 3 shows dose-response curves and combined data. Model parameters are shown in the bottom row of Table 1.

**Figure 4:**
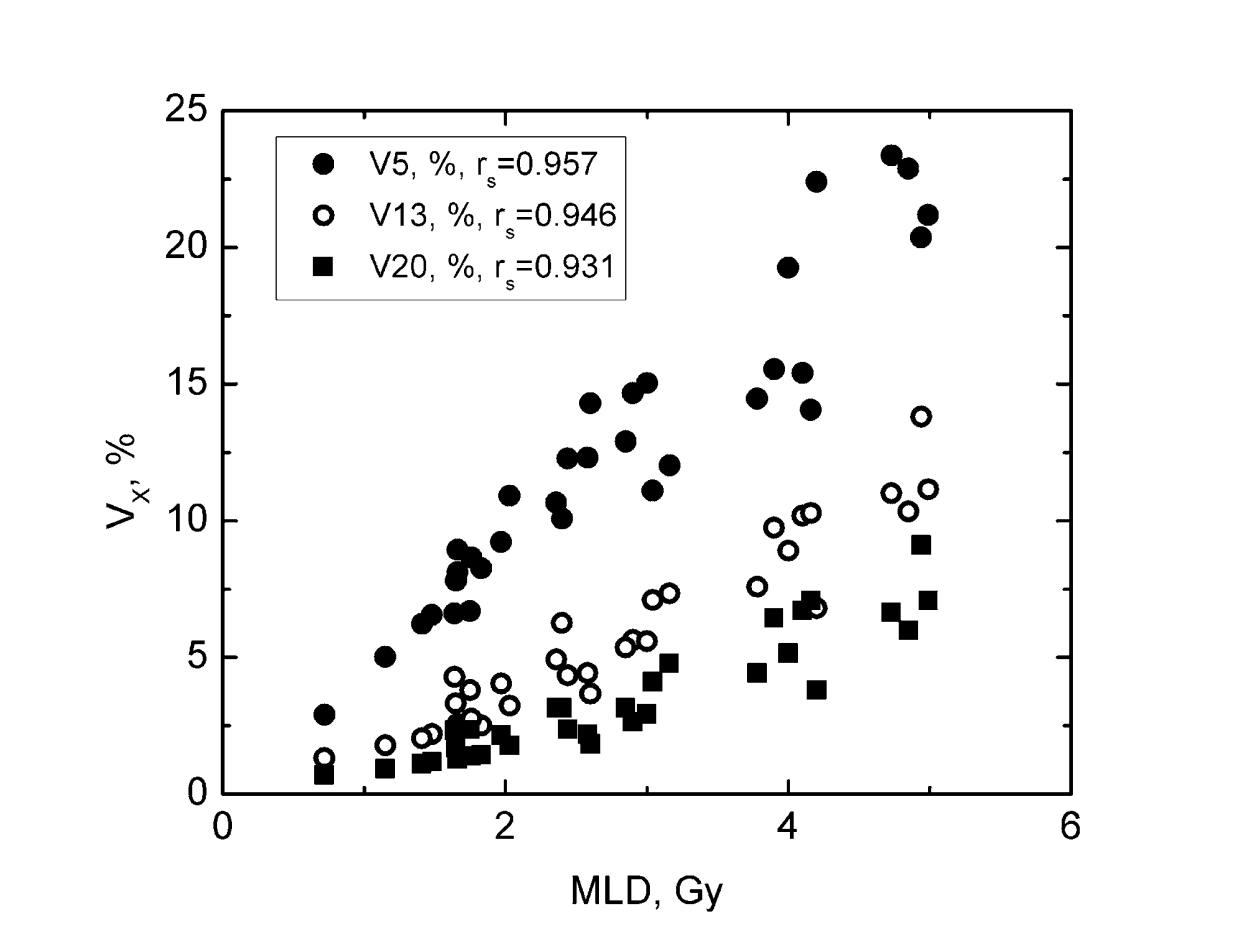
Scatterplot showing correlations between mean lung dose (MLD) and V5, V13 and V20. Insert shows Spearman’s rank-order correlation coefficients.

Table 2 shows the correlations between the quantity in the “Parameter” column with normal lung MLD and V_20_; the latter has been reported most broadly in the literature [10]. Spearman’s rank-order correlation coefficients showed that both V_5_ and V_10_ strongly correlate with MLD in cohort A, Rs = 0.936 and 0.966, respectively. Overall, there was statistically significant correlation between all Vx as a percent volume and MLD, as well as between V_x_ and V_20_. Correlation was weaker for tumor-specific parameters and insignificant for PTV D_95_, cohort B. The correlations were also tested in the UCSD independent validation cohort. Figure 4 shows strong correlation between V_5_, V_13_ ,V_20_ and MLD for the UCSD cohort with large and significant Spearman’s rank-order correlation coefficients, (Rs ≥ 0.931, p < 0.001). Effect of possible confounders, specifically pulmonary emphysema, tumor location (upper/middle vs lower) and ILD was studied in the original report for the cohort A [21]. ILD was the only factor significantly associated with RP. In the cohort B [22] one patient had COPD, four had emphysema, tumor location was peripheral in 21 of 25 patients and peripheral/central in remaining four. This is insufficient to perform statistical analyses to assess effect of possible confounders.

**Table 2:** Spearman’s rank-order correlation coefficients for the considered tumor and lung-specific parameters. * 0.01 ≤ p < 0.05; ** 0.001 ≤ p < 0.01; *** p < 0.001

Cohort A [21]			Cohort B [22]		
Parameter	MLD, Gy	Lung V_20_, %	Parameter	MLD Gy	V_20_
GTV, cc	0.643^***^	0.667^***^	ITV, cc	0.583^**^	0.654^***^
PTV, cc	0.623^***^	0.650^***^	PTV, cc	0.556^**^	0.627^***^
GTV D_95_, Gy	0.376^**^	0.398^***^	PTV D_95_ Gy	0.053	0.169
V_5_, %	0.936^***^	0.783^***^	V_20_, %	0.764^***^	-
V_10_,%	0.966^***^	0.914^***^	V_40_, %	0.621^***^	0.679^***^
V_20_, %	0.927^***^	-	V_45_, %	0.593^**^	0.612^**^
V_30_,%	0.914^***^	0.984^***^	MLD Gy	-	0.764^***^
MLD, Gy	-	0.927^***^	V_24_, cc	0.514^**^	0.602^**^
			V_48_, cc	0.487^*^	0.476^*^

Incidence of radiation-induced lung toxicity as a function of MLD was separately assessed for patients with and without underlying interstitial lung disease (ILD). In Cohort A, 11 patients had subclinical ILD before SBRT as did at least three in Cohort B. However, because of the small number of patients with toxicities, best values were only obtained for patients with ILD in the Cohort A, grade 2+ toxicity. While 95% CI were not established, it was possible to calculate 68% CI. Obtained logistic model parameters were indeed indicative of substantially higher sensitivity, D_50 _= 3.7 Gy (68% CI 2.7-5.1 Gy), and γ_50 _= 0.73 (0.22-1.34) as opposed to D_50_ of 6.1 Gy, γ_50_ of 1.18 for combined cohort, Grade 2+ model. This observation, however, must be interpreted with caution as the dose-response was not statistically significant.

## Discussion

Developing evidence-based dose-volume metrics to generate guidelines for normal tissue sparing is a challenging task assigned to working groups, such as QUANTEC [1], HyTEC [2, 3] or PENTEC [5]. Selected metrics have to be justified on statistical and mechanistic merits and hypothesis-generating studies taking advantage of detailed patient-specific data provide this justification. Currently, both Vx and MLD are commonly used dosimetric correlates for lung toxicity following SBRT [2, 19]. Supplementary Table 1 in the HyTEC report on lung toxicity following SBRT [2] shows 16 publications where lung toxicity correlated with either V_5_, V_10_, V_13_, V_20_ or MLD, with V_20_ and MLD being the most popular choices attempted in 15 publications.

In considering tumor- and target-specific (e.g., GTV, PTV) and normal lung-specific (e.g., MLD, V_x_) metrics, our results showed that MLD was the only parameter that consistently correlated with incidence of RP across both cohorts A and B, as well as in the combined cohort. Proponents for MLD argue that regional lung function is compromised at any dose, with the underlying assumption that local injury is linearly dependent on dose [24]. Local dose-response for perfusion loss has been demonstrated in SBRT patients [25]. Studies of changes in regional lung perfusion as a function of conventional fractionated doses have shown that this dependence may follow a sigmoid relationship, with no loss of perfusion below 10-15 Gy and a plateau at about 55 Gy [24]. Although this deviates from the linear dependence theory, the authors deemed MLD as a reasonable surrogate for overall response parameter, which is a sum of regional injuries. This implies mechanistically-based use of MLD in SBRT and emphasizes the need to understand the local lung function (ventilation and perfusion) before SBRT. In theory, if there is a threshold dose beyond which function is 100% compromised, it will be lower for SBRT because using Radiation Biologically Effective Dose (BED) Calculator, 60Gy/30 has BED(3) of 100Gy, which is the same BED(3) of 31.9Gy/5, assuming α/β = 3Gy.

Those who advocate for Vx argue that lung regions are incapacitated after receiving above a certain threshold dose and that toxicity is avoided if a minimum total lung volume is undamaged. Unfortunately, data for conventional fractionation argues against the existence of a single, or even narrowly distributed threshold. Indeed, the QUANTEC report showed a broad range in threshold doses (V_5_ to V_40_) correlating to incidence of RP while also noting two important findings: 1) different V_x_ parameters correlate with one another and MLD; 2) use of V_x_ cut-offs has to be noticed as they may not be transferrable between radiotherapy techniques [19]. Supporting this first finding, we also saw significant correlation between V_5_, V_13_ ,V_20_ and MLD, p < 0.001, in our independent cohort. While this makes it impossible based on statistical analysis alone to single out the best predictor, it also means that, at least in a single institution setting, using MLD is as good as any other metric. Advanced statistical methods, for example Lasso or elastic nets regularized regression allow us to address the problem of co-linearity. This complex problem which requires large sets of detailed data has been gaining attention in the literature.

Robustness of MLD may be questioned because dose alone is insufficient to describe biological effect and accounting for dose-per-fraction may be needed, in particular for normal tissues. Notably, in this study physical dose without corrections for dose-per-fraction such as biologically equivalent dose in 2 Gy fractions (EQD2), was used for both the dose in V_x_ and MLD. MLD was the strongest predictor despite a broad range in the number of fractions in the two cohorts. This provides the simplest way to guide treatment planning but also raises a question as to whether this is also a consequence of statistical correlation. Specifically, if MLD defined as physical dose and mean dose calculated following conversion into EQD2 are correlated, finding preferred metric will be challenging. In this study we were restricted to what was reported. Conversion of MLD to mean EQD2 is non-linear and cannot be performed without full DVH data. This is a limitation in our study and conversion to common fractionation, for example EQD2 is encouraged.

Variations of the volume cut-off approach, specifically absolute volume spared, have been put forward [26] and these may provide connection with a mechanistic nature of development of RP. For example, lung dose-thresholds used in the RTOG 0915 trial required that less than 1500 and 1000 cc of total lung (minus PTV) receive a specified dose, to minimize risks of pulmonary function decline and pneumonitis, respectively. Similar thresholds were included in the American Association of Physicists in Medicine Task Group 101 report, which they describe as “minimum critical volume below threshold” [26]. Spared volume style constraints have been used for other parallel tissues, such as liver [3], though further investigation of their validity is needed.

Considering biological mechanisms, published studies propose various pro-inflammatory cytokines that contribute to the acute inflammation and late fibrosis seen in radiation pneumonitis. This inflammatory cascade has several well-recognized triggers including DNA double-strand breaks, endothelial cell damage, free radical damage, upregulation of leukocyte adhesion molecules and platelet derived growth factor (PDGF) isoforms, activation of type II pneumocytes, and hypoxia [27, 28]. The existence of several cascade triggers supports the theory that inflammation follows a linearly dose dependent response rather than the incapacitation hypothesis underlying the V_x_ approach. On this basis, use of MLD is supported as it fundamentally parallels the biological inflammatory dose-response.

Decline in pulmonary function after SBRT is a potential risk, though the extent to which this occurs is unclear. As some studies suggest no or minimal decline in pulmonary function, and tumor progression can be life threatening, baseline pulmonary functional status may not be a major factor in decision making with respect to SBRT dose-fractionation. With some studies suggesting a progressive decline in diffusion capacity, risk of pulmonary function decline should be discussed with the patient, though such changes can occur as part of the natural course of chronic obstructive pulmonary disease (COPD). In patients without COPD, diffusion capacity could potentially improve after SBRT. In contrast, patients with interstitial lung disease, regardless of measured pulmonary function, are at high risk of life-threatening toxicity after SBRT [2], and these risks must be weighed against potential benefits. Deferring treatment, or use of more stringent lung dosimetry should be considered.

Patient- and disease-specific parameters, for example age, sex, tumor location, performance status, may also play a role in development of RP [2]. Effect of age, sex, tumor location and performance status were investigated for correlation with RP in one of the source publications [21]. None of these factors were statistically significant with respect to predicting RP risks. Notably, another study [29] demonstrated that the incidence of RP can be minimized if patients are screened for radiographic evidence of ILD [15, 22] and high values of biological markers (KL-6 and SP-D) before treatment. Increased treatment-related toxicity in patients with co-existing interstitial lung disease who receive SBRT has been noted in a comprehensive review [30]. Small sample size as well as relatively low toxicity rates were further limitations of our study. This, unfortunately, is a common problem with outcomes data reporting. Detailed patient-specific data are typically not available for large cohorts.

## Conclusions

Our findings show that there is a strong statistical basis for using MLD as a predictor of RP within single institutions. These findings are also supported by mechanistic considerations. The relatively simple MLD metric has implications for clinical practice and lung SBRT clinical trials, especially in combination with immunotherapy (trials NCT03867175 and NCT03436056). While validation is required with data from different institutions, MLD is promising for use across institutions and in cooperative group trials. Limiting bilateral MLD <8 Gy was recommended in the HyTEC report for fractionated SBRT to ensure safety. Our study provides further justification for using MLD to predict RP risks. Competing Vx and critical volume (i.e., spared lung) approaches may have promise, but need further validation. Our main conclusion is simply that MLD may have merit for SBRT, and it should at least be considered along with the more common V_x_ and critical volume metrics in future studies.
